# Supplementary material for: A methodology for the design of experiments in computational intelligence with multiple regression models
Source: PeerJ. 2016 Dec 1;4:e2721. doi: 10.7717/peerj.2721 (PMC5136129; doi:10.7717/peerj.2721)
Supplement: Data S1 — Datailed results from UC Irvine Machine Learning Repository (Housing, Machine CPU, Wine Quality, Automobile and Parkinson) and the 3 Use Cases (Protein Corona, Gajewicz Metal Oxides and Aquatic Toxicity) [file peerj-04-2721-s001.zip › 6_UseCases/1_ProteinCorona/DifModels.RMSE.iSplits.6.pdf]

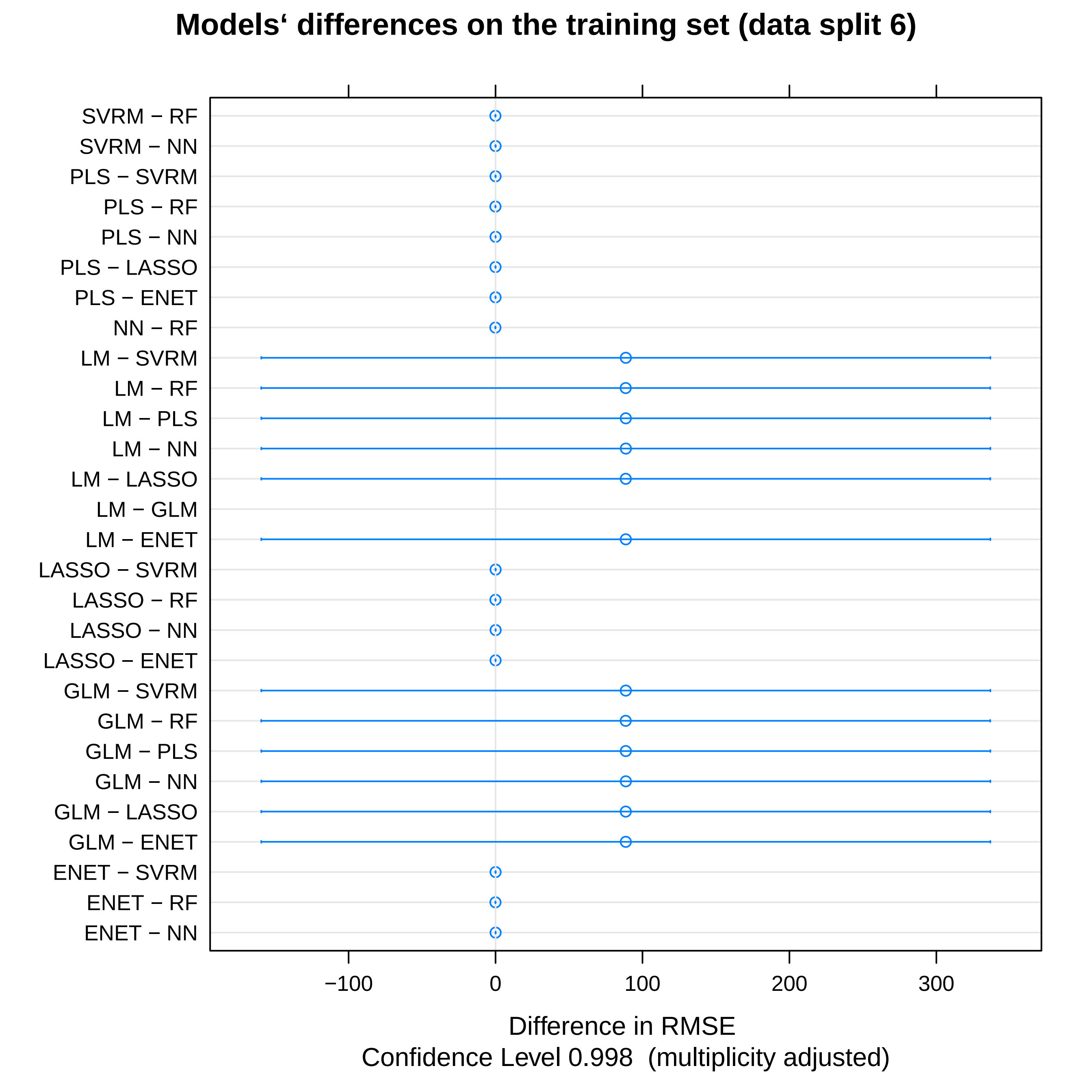

Models‘ differences on the training set (data split 6)
SVRM − RF
SVRM − NN
PLS − SVRM
PLS − RF
PLS − NN
PLS − LASSO
PLS − ENET
NN − RF
LM − SVRM
LM − RF
LM − PLS
LM − NN
LM − LASSO
LM − GLM
LM − ENET
LASSO − SVRM
LASSO − RF
LASSO − NN
LASSO − ENET
GLM − SVRM
GLM − RF
GLM − PLS
GLM − NN
GLM − LASSO
GLM − ENET
ENET − SVRM
ENET − RF
ENET − NN
−100
0
100
200
300
Difference in RMSE
Confidence Level 0.998 (multiplicity adjusted)
